# Ultrasensitive and visual detection of human norovirus genotype GII.4 or GII.17 using CRISPR-Cas12a assay

**DOI:** 10.1186/s12985-022-01878-z

**Published:** 2022-09-17

**Authors:** Weidong Qian, Jie Huang, Ting Wang, Cheng Fan, Jie Kang, Qian Zhang, Yongdong Li, Si Chen

**Affiliations:** 1 School of Food and Biological Engineering, Shaanxi University of Science and Technology, Xi’an, 710021 People’s Republic of China; 2 Shaanxi Testing Institute of Product Quality Supervision, Xi’an, 710048 People’s Republic of China; 3 Department of Dermatology, Huazhong University of Science and Technology Union Shenzhen Hospital, Shenzhen, 518052 People’s Republic of China; 4 Ningbo Municipal Center for Disease Control and Prevention, Ningbo, 315010 People’s Republic of China; 5 Shenzhen University Health Science Center, Shenzhen, 518060 People’s Republic of China

**Keywords:** CRISPR-Cas12a, RT-RAA, Diagnostics, Human norovirus, GII.4 or GII.17

## Abstract

**Background:**

Integrating CRISPR-Cas12a sensors with isothermal signal amplification can be exploited to develop low-cost, disposable, and ultrasensitive assays for the diagnostics of human pathogens.

**Methods:**

RT-RAA-Cas12a-mediated real-time or end-point fluorescent and lateral flow strip (LFS) assays for direct detection of norovirus (NOV) genotype GII.4 or GII.17 were explored.

**Results:**

The results showed that our RT-RAA-Cas12a-mediated fluorescent and LFS assay could detect NOV GII.4 or GII.17 by targeting the viral protein 1 gene. Our RT-RAA-Cas12a-mediated fluorescent and LFS assay can specifically detect NOV GII.4 or GII.17 with no cross-reactivity for other related viruses. The low limit of detection could reach 0.1 copies/μL within approximately 30–40 min, and the results were visualized using an ultraviolet light illuminator or on a LFS without complex equipment. In addition, our RT-RAA-Cas12a-mediated fluorescent and LFS assay provided a visual and faster alternative to real-time RT-PCR assay, with 95.7% and 94.3% positive predictive agreement and 100% negative predictive agreement.

**Conclusions:**

Together, our RT-RAA-Cas12a-mediated approach would have a great potential for point-of-care diagnostics of NOV GII.4 and/or GII.17 in resource-limited settings.

## Introduction

Human noroviruses (NOVs) are now recognized as a leading cause of the majority of all non-bacterial gastroenteritis [1]. NOVs are a group of RNA viruses that can cause symptoms such as acute vomiting and diarrhoea generally lasting for 48 h in both otherwise healthy children and adults. NOVs are highly infectious, as even a few particles can cause disease, and infected individuals shed high loads of virus [2, 3]. NOVs are mainly transmitted to humans through exposure to contaminated foods and water as a result of direct or indirect contact with NOV-infected human faeces [4], or within aerosols that are generated by vomiting from infected individuals [5]. As a result of the high infectivity of NOVs and their ability for efficient transmission, outbreaks of NOVs have been reported globally in closed community settings including hospitals and care homes, and thus require intervention measures for reduction in aggressive infection [6].

Human noroviruses hold immense genetic diversity with ten different genogroups (GI-GX) and at least 48 identified genotypes [7]. Genogroups GI, GII and GIV of NOVs are known to be associated with human infection. Among about 21 genotypes of the NOV GII, the genotype GII.4 has been responsible for the majority of clinical cases of breakthrough infection of NOVs in the past decade [8]. Recently, a novel genotype of GII.17 of NOVs has emerged and spread rapidly to become the dominant strain of NOVs in some parts of Asia, posing the risk of further threats of outbreaks [9]. Until now, apart from only a few reports on effective antiviral treatment in humans [10], there is currently no licensed NOVs vaccine for humans available [11]. As a result, a more rapid and early detection of NOVs infection plays a significant role in facilitating early intervention, treatment and infection prevention, which, in turn, may mitigate the transmission risk of the infectious virus.

The current gold standard for detecting NOVs is molecular techniques including reverse transcription-polymerase chain reaction (RT-PCR), nested RT-PCR, and real-time RT-PCR [12]. Among those methods, real-time RT-PCR has been extensively employed to detect or diagnose NoV infection due to its high sensitivity and specificity, as well as low-risk of carry-over contamination. Currently, various commercially available real-time RT-PCR assays demonstrated the sensitivity of real-time RT-PCR at 10–50 genome copies/reaction for NoV GI and 1–300 genome copies/reaction for NoV GII [13]. However, real-time RT-PCR method typically relies on sophisticated equipment and highly-skilled personnel, and has an average reaction time of ~ 2 h, all of which is not suitable for simple, rapid, and point-of-care (POC) molecular assay for diagnosing NOVs infection in resource-constrained areas for routine detection. In a recent study, Sun et al. presented a paper-based colorimetric method for detecting and distinguishing the GII.4 and GII.17 genotypes of NOVs [14]. However, the test by using this method needs a relatively long time (~ 3 h), and the detection range is limited between 2.6 fM and 0.5 pM, and is thus less sensitive compared with RT-PCR [14]. Conversely, due to the excellent rapidity, sensitivity and specificity, RNA-guided clustered nuclease-based nucleic acid detection system consisting of the regularly interspaced short palindromic repeats (CRISPR) and their CRISPR-associated (Cas) nucleases, has recently shown considerable potential to exploit next-generation POC molecular diagnostics for infectious pathogens [15–17]. Currently, the efficacy of several versions of Cas nucleases including Cas12a, Cas12b, Cas13a, and Cas14, has been evaluated in both in vitro and in vivo assays [18–21]. Among these nucleases, Cas12a (formerly Cpf1) is a class II type V endonuclease. Cas12a containing a RuvC nuclease domain is ushered by a single CRISPR RNA (crRNA) containing a T-rich protospacer-adjacent motif (PAM) sequence to cleave double-stranded DNA (dsDNA) at specific site, or without PAM to perform nonspecific ssDNA cleavage in trans in vitro [18]. Moreover, combining Cas12a nucleases with recombinase polymerase amplification (RPA) or reverse transcription-RPA (RT-RPA) has been thoroughly explored to develop DNA Endonuclease-Targeted CRISPR Trans Reporter (DETECTR) system and significantly enhances sensitivity and specificity of nucleic acid detection [18].

In this study, by targeting the viral protein 1 (VP1) gene of genotype GII.4 and GII.17 of HNOVs, a combination of reverse transcription recombinase-aided amplification (RT-RAA) with CRISPR-Cas12a, designated as RT-RAA-Cas12a, is capable of detecting few copies of nucleic acids (RNA) with a much higher degree of specificity without the need of high-cost instruments. In addition, our RT-RAA-Cas12a-mediated fluorescent and lateral flow strip (LFS) method were further validated by testing 80 clinical specimens and achieved highly consistent results with those of conventional RT-PCR method. In summary, we demonstrate a CRISPR-Cas12a-mediated assay for rapid, ultrasensitive, specific, and visual, instrument-free POC detection of NOVs genotype GII.4 or GII.17.

## Materials and methods

### Clinical samples

A total of 80 archived stool specimens with Ct values under 30 (< 30 Ct) were collected from individuals displaying NOV-related clinical symptoms, among which 40 and 30 samples were tested as positive for NOV genotype GII.4 and GII.17, respectively, and 10 samples, including GII. 2, GII. 3 and GII. 6 according to a previous method [22]. These samples used in this study were collected by Ningbo Municipal Center for Disease Control and Prevention (NCDC) between January, 2016 and December, 2020. Ethical approval for this study was obtained from the ethical committee of NCDC.

### Reagents

FAM-TTATTATT-quencher (ssDNA FQ), FAM-TTATTATT-biotin (ssDNA FB) and probes were generated by GENEWIZ Inc. (Suzhou, China). Oligonucleotides of all the primers employed in this study were synthesized by Sangon Biotech (Shanghai, China), and displayed in Table 1. RT-RAA nucleic acid amplification kit was purchased from Jiangsu Qitian Gene Biotechnology Co., Ltd (Wuxi, China). NEbuffer 2.1 and Cas12a were purchased from New England Biolabs (MA, USA). The LFS was purchased from Tiosbio (Nanjing, China).

**Table 1:** The oligonucleotides used for RT-RAA and crRNA in RT-RAA-Cas12a-based assay for norovirus genotype GII.4 and GII.17 detection

Assay	Name	Oligonucleotide sequences (5′-3′)
RT-RAA	NOV-F1	GTGCCCAGACAAGAGYCAATGTTCAGATGGATG
	NOV-F2	CAATGTTCAGATGGATGAGRTTCTCAGATC
	NOV-R1	ACCATTGTACATYCTKGMCAAATGAG
	NOV-R2	ATGAGMMARRTARGGATTCAAATCAGGGCC
Cas12-based detection	crRNA1-F	GAAATTAATACGACTCACTATAGGGTAATTTCTACTAAGTGTAGATAGATTGCGATCGCCCTCCCA
	crRNA1-R	TGGGAGGGCGATCGCAATCTATCTACACTTAGTAGAAATTACCCTATAGTGAGTCGTATTAATTTC
	crRNA2-1-F	GAAATTAATACGACTCACTATAGGGTAATTTCTACTAAGTGTAGATTTGTGAATGAAGATGGCGTC
	crRNA2-1-R	GACGCCATCTTCATTCACAAATCTACACTTAGTAGAAATTACCCTATAGTGAGTCGTATTAATTTC
	crRNA2-2-F	GAAATTAATACGACTCACTATAGGGTAATTTCTACTAAGTGTAGATTGAATGAAGATGGCGTCGA
	crRNA2-2-R	TCGACGCCATCTTCATTCAATCTACACTTAGTAGAAATTACCCTATAGTGAGTCGTATTAATTTC
	crRNA3-F	GAAATTAATACGACTCACTATAGGGTAATTTCTACTAAGTGTAGATTTCTAATCCAGGGGTCAATT
	crRNA3-R	AATTGACCCCTGGATTAGAAATCTACACTTAGTAGAAATTACCCTATAGTGAGTCGTATTAATTTC

### Production of standard RNA of NOVs genotype GII.4 and GII.17

**Fig. 1:**
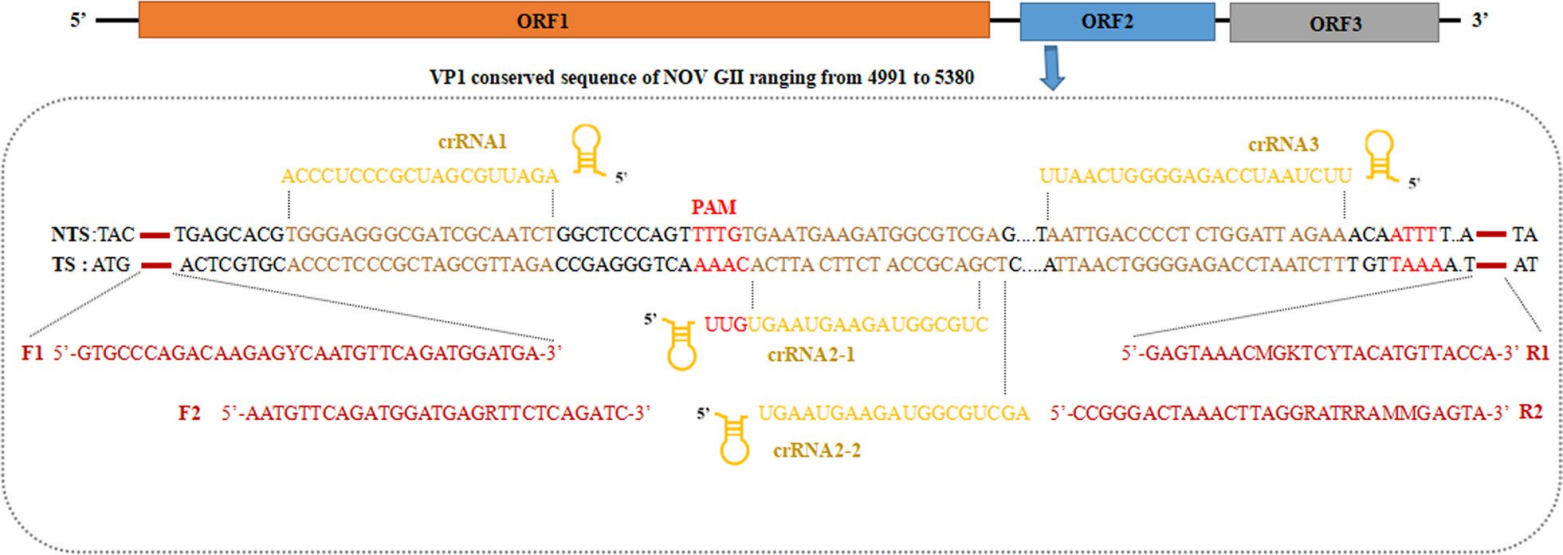
Visualization of primers for reverse transcription recombinase-aided amplification (RT-RAA) and crRNA spacer sites in the target viral protein 1 (VP1) gene sequence of NOV genome. RT-RAA primers are indicated by red rectangles. crRNAs are programmed to specifically target VP1 gene of noroviruses (NOV) GII.4 and GII.17

Whole genome sequences of different strains of NOVs genotype GII.4 or GII.17 were recovered from the NCBI database and aligned using ClustalW. Based on the alignment result, a specific consensus region of viral protein 1 (VP1) gene corresponding to sequences 4991–5380 was determined as the target (Fig. 1). The DNA fragment of the target sequence was synthesized and inserted into plasmid pBluscript II SK (+) (Sangon, Shanghai, China) (Fig. 1). The resulting pBluscript-NOV was transformed into *Escherichia coli* TOP10 cells to construct the recombinant strain TOP-NOV. Then, the pBluscript-NOV was extracted using the TIANprep Mini Plasmid Kit (Tiangen Biotech, Beijing, China), and linearized with *Sac* I. The linear pBluscript-NOV was employed as the template of the in vitro transcription (IVT) reaction to generate NOV RNA standard using IVT T7 Kit (TaKaRa, Dalian, China). The IVT reaction mixture was composed of 5 μL of 10 × transcription buffer, 5 μL of each NTP solution, 1 μL of RNase inhibitor, 5 μL of T7 RNA polymerase, 6.5 μL of RNase-free water and 12.5 μL of linear pBluscript-NOV plasmid, and incubated at 39 °C for 2 h. Finally, RNA concentration in nanogram was converted to that of RNA copy number using the following formula: RNA copy number = [M (ng/μL) × 6.02 × 10^23^]/(N × 10^9^ × 340), where M refers to the RNA concentration quantified by a spectrophotometer (Metash Instruments, Shanghai, China), N refers to the length of RNA.

### Design and preparation of crRNAs

Four crRNA with different nucleotides of crRNA spacer sequences targeting the highly conserved region of the VP1 gene were determined, and further evaluated for the specificity of each crRNA to NOVs genotype GII.4 or GII.17 using the Basic Local Alignment Search Tool. Moreover, the 21 nucleotides of 5′-TAATTTCTACTAAGTGTAGAT-3′ were used for crRNA stem sequence, and served as a binding scaffold for Cas12a. Preparation of crRNA was performed using the following step. The oligonucleotides for the production of crRNA preparation listed in Table 1 were chemically synthesized by GENEWIZ. (Suzhou, China), and annealed. The resulting double strand DNAs (dsDNA) were further transcribed by in vitro transcription using IVT T7 Kit. The transcription reaction comprised 5 μL of 10 × transcription buffer, 5 μL of each NTP solution, 1 μL of RNase inhibitor, 5 μL of T7 RNA polymerase, 9 μL of RNase-free water and 10 μL of annealed dsDNA, and incubated at 39 °C for 2 h. The resulting crRNA products were purified using phenol–chloroform extraction and isopropanol precipitation, and the concentration of crRNA was determined using a spectrophotometer.

### Optimization of reaction condition of RT-RAA

The performance of RT-RAA was evaluated by comparing three factors consisting of different primers, the specificity, and the sensitivity of the assay. The RT-RAA reaction mixture comprised 25 μL of reaction buffer V, 2 μL of each primer (10 μM), 16.5 μL of ddH_2_O, 2 μL of standard RNA and 2.5 μL of 280 mM magnesium acetate. Then the reaction tubes were placed into the preheated Axxin T8 isothermal instrument for 20 min at 39 °C. Subsequently, the RT-RAA products were treated with an equal volume of 50 μL phenol/chloroform, subjected to agarose gel electrophoresis (2%) and finally evaluated under an ultraviolet (UV) light.

### Optimization of RT-RAA-Cas12a-mediated fluorescent and LFS assay

The RT-RAA-Cas12a-mediated assay was carried out using 5 μL of RT-RAA products in a total reaction volume of 50 μL, as well as 45 μL of the CRISPR-Cas12a reaction mixture, which contained 5 μL of 10 × NEBuffer 2.1, 1 μL of 100 nM crRNA, 1 μL of 1 μM Cas12a, 0.5 μL RNase inhibitor (40 U), and 2 μL of 1 μM ssDNA reporters, 35.5 μL of RNase-free water. Then, the reactions were conducted in the preheated Axxin T8 isothermal instrument for 15 min at 39 °C with fluorescent signals collected every 10 s (ssDNA FQ substrates = λex: 485 nm; λem: 535 nm), and visualized by a UV light illuminator and LFS with ssDNA FB as the substrates.

### Specificity of RT-RAA-Cas12a-mediated fluorescent and LFS assay

The specificity of RT-RAA-Cas12a-mediated assay was performed by testing each RNA samples at a concentration of 200 copies/μL from other gastrointestinal viruses, including human rotavirus (HRV), astrovirus (HAtV), and enterovirus 71 (HEV71). 10 different samples testing positive for each test virus as well as 10 non-GII.4 or GII.17 samples were employed as the control in this study.

### Sensitivity of RT-RAA-Cas12a-mediated fluorescent and LFS assay

To evaluate the sensitivity of RT-RAA-Cas12a-mediated assay, a concentration gradient of RNA standard samples, which was adjusted to be 200, 100, 10, 1, 0.5, 0.1 and 0.05 copies/µL, was used as the template in RT-RAA reaction. All volumes of Cas12a-mediated assay were 50 µL as described above. Each reaction process was replicated three times and the results were analyzed by fluorescence and LFS.

### RT-PCR for clinical samples

The total RNA of 80 samples, each 200 µL of clinical solution, was manually extracted using BeaverBeads™ Viral DNA/RNA Kit BEAVER, Suzhou, China), and automatically performed using automatic nucleic acid extraction instrument (bioPerfectus technologies, Jiangsu, China), respectively. The RT-PCR detection for NOV nucleic acids of clinical samples was performed using NOV test kit (bioPerfectus technologies, Jiangsu, China) in an ABI 7500 (Applied Biosystems). The reactions were performed with an initial step of reverse transcription at 48 °C for 30 min, followed by 95 °C for 15 s, 35 cycles of 95 °C for 15 s, and 53 °C for 1 min.

### Statistics

Each sample was conducted in at least three independent biological replicates. Statistical analysis of the data involving end-point fluorescence was performed using the Prism 8 (GraphPad Software, version 8.0.1) and statistical differences were assessed by the Students’ *t*-test. The unpaired twotailed t-test was applied to investigate the differences between groups and the threshold for defining significance was based on the *p* value < 0.05.

## Results

### Screening of optimal RT-RAA primers and crRNA guides

**Fig. 2:**
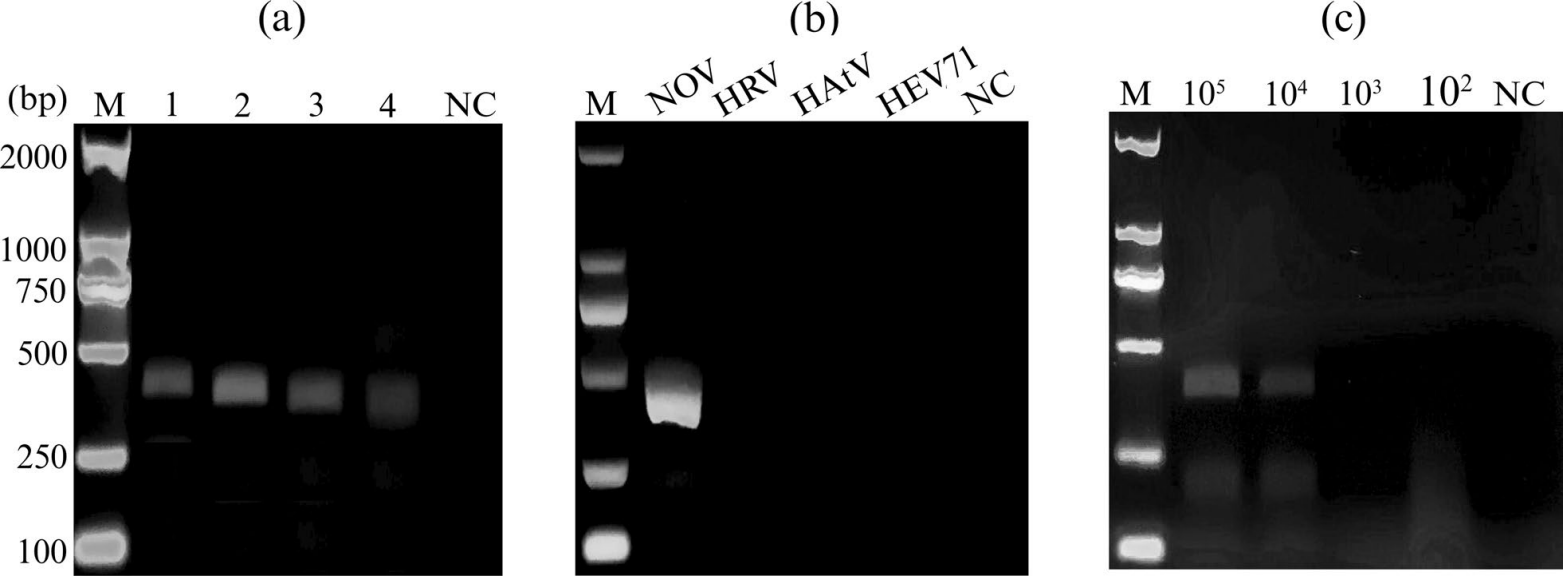
Assessment of primer pairs for reverse transcription recombinase-aided amplification (RT-RAA). **a** The optimal primer pairs for RT-RAA were evaluated with RNA standard of NOV GII.4 or GII.17 at 1 × 10^5^ copies/μL, respectively. Lane 1: NOV-F1/NOV-R1; Lane 2: NOV-F2/NOV-R1; Lane 3: NOV-F1/NOV-R2; Lane 4: NOV-F2/NOV-R2. **b** Specificity of RT-RAA assay was conducted with three gastrointestinal viruses, including human rotavirus (HRV), astrovirus (HAtV), and enterovirus 71 (HEV71), and the results were demonstrated by agarose electrophoresis. NC, negative control. **c** Sensitivity of RT-RAA assay was performed using the concentration of RNA standard of NOV GII.4 and GII.17 ranging from 1 × 10^5^ to 1 × 10^2^ copies/μL

We first optimized RT-RAA primers for RT-RAA assay because the optimal primer pair played a crucial role in high productivities of the dsDNA under optimized conditions. The highly conserved region of the VP1 gene of NOVs genome, was determined as the crRNA target sites based on the alignment of reported NOVs GII.4 and GII.17 genomes, which showed 92.95% sequence homology. In this study, based on the sequence of VP1 gene, different primer pairs and crRNA were designed to select optimal primer pairs and crRNA (Fig. 1). The amplification efficiency of four primer pairs was investigated and compared at RNA standard concentrations of 1 × 10^5^ copies/μL at 39 °C for 20 min using RT-RAA kit. As demonstrated in Fig. 2a, only NOV-F2/NOV-R1 primer pairs generated clear bands with expected size of 369 bp at 1 × 10^5^ copies/μL. Subsequently, NOV-F2/NOV-R1 specificity was validated using other viral RNA samples as the template with the concentration of 1 × 10^5^ copies/μL. Figure 2b indicated that the NOV-F2/NOV-R1 primer pairs did not cross react with other relevant viruses including HRV, HAtV, and HEV71. Moreover, serial dilutions of the RNA standard ranging from 1 × 10^2^ to 1 × 10^5^ copies/μL were applied to evaluate the sensitivity of RT-RAA reaction. Figure 2c showed that NOV-F2/NOV-R1 produced a single and clear band at concentrations of 1 × 10^4^ copies/μL. From these data, we concluded that NOV-F2 and NOV-R1 was the most efficient primer pair, and thus was employed for the subsequent experiments.

**Fig. 3:**
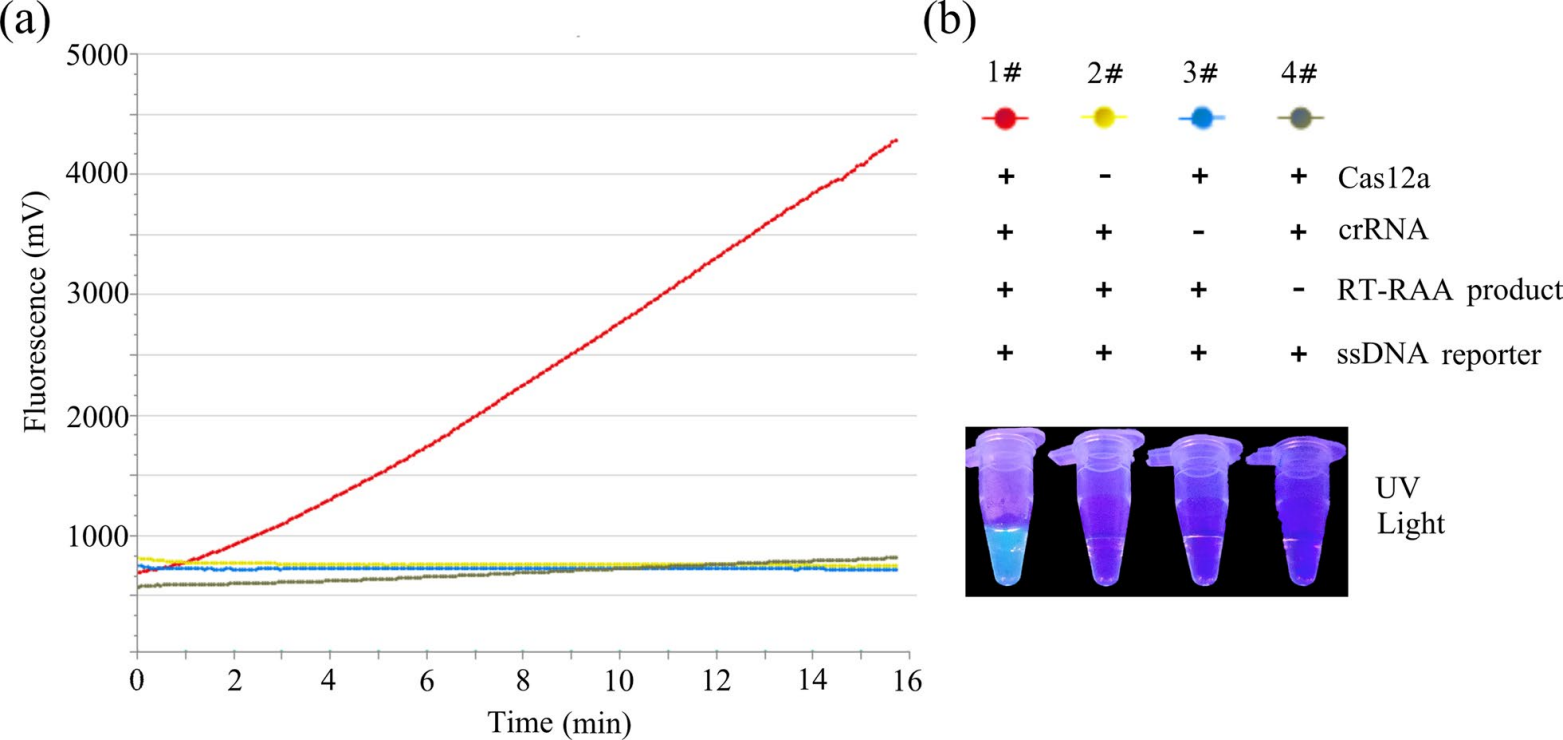
Feasibility analysis of RT-RAA-Cas12a-based assay for NOVs GII.4 or GII.17 detection. Real-time (**a**) and end-point (**b**) fluorescence of RT-RAA-Cas12a-based assay was examined in the presence or absence of basic reagents at 39 °C for 30 min. The images of the tubes were taken under ultraviolet (UV) light after 30 min incubation

To evaluate the feasibility of RT-RAA-Cas12a-mediated real-time or end-point fluorescence assay for the detection of NOVs GII.4 or GII.17, four basic reagents of the assay were identified: crRNA, Cas12a, target DNA, and ssDNA FQ reporters. Four reaction systems with various reagents were prepared and investigated. After 20 min of incubation at 39 °C, only the reaction system including RT-RAA products, crRNA, Cas12a, and ssDNA FQ reporters generated a bright fluorescence signal (Fig. 3a), where the fluorescence intensity enhanced continuously with the increasing cleavage number of ssDNA FQ reporters. Notably, a color change from blue to green was observed in the reaction tube # 1 when illuminated with a UV light illuminator (Fig. 3b).

**Fig. 4:**
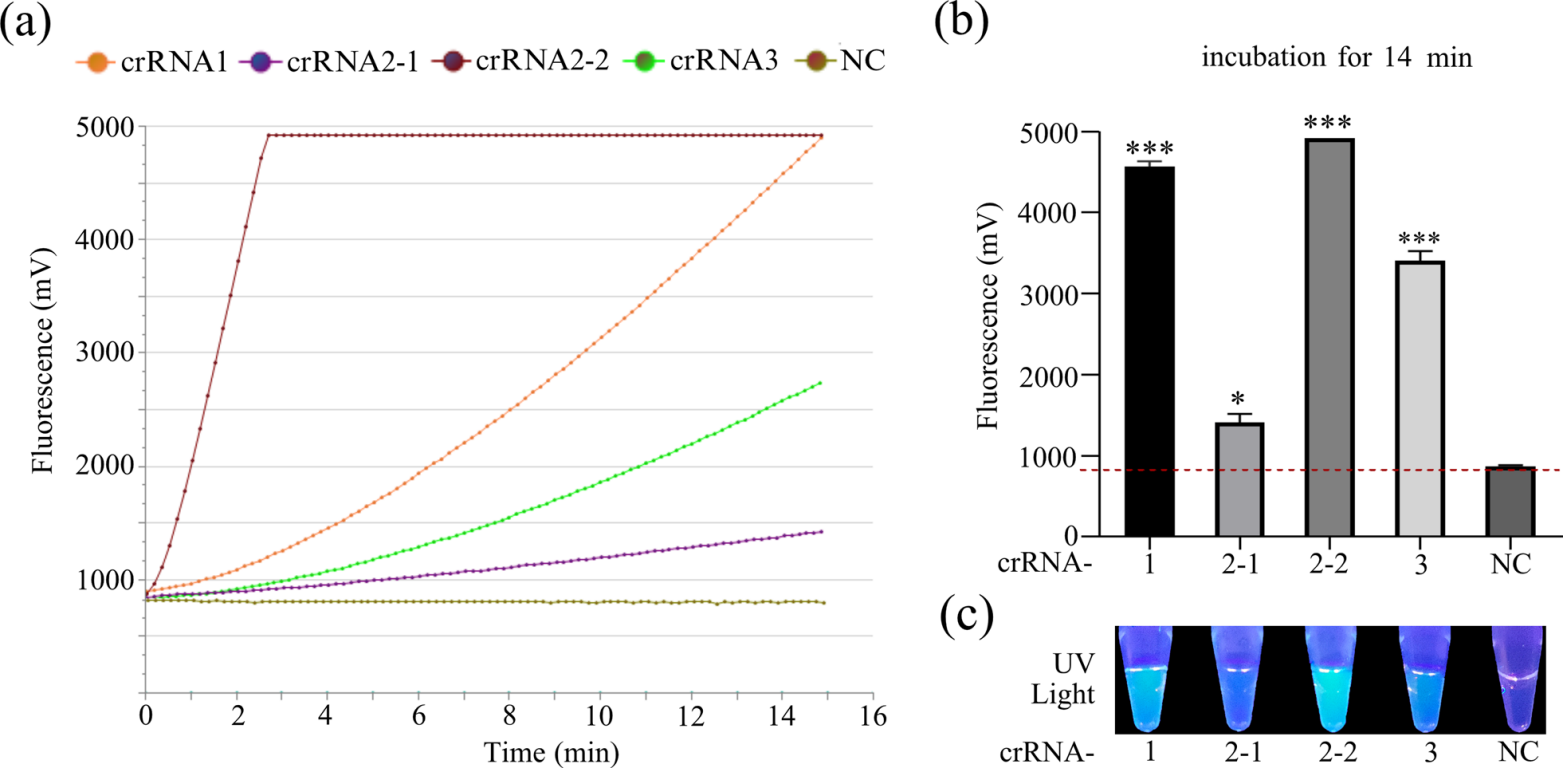
Screening of optimal crRNA for RT-RAA-Cas12a-based assay. The RT-RAA-Cas12a-based real-time (**a**) and end-point (**b**) fluorescence readouts were measured after incubation with 30 nM of different crRNA for 15 min and 14 min at 39 °C, respectively. Three replicates were performed for each sample. Error bars refer to the standard deviations at three replicates (n = 3). Statistical analysis was applied to evaluate the difference between test groups to NC. *p < 0.05; **p < 0.01; ***p < 0.001. NC, negative control. **c** Visualization of RT-RAA-Cas12a-based detection under ultraviolet (UV) light

The appropriate crRNA facilitated base pairing of the crRNA seed with the target strand of dsDNA, yielding high fidelity and sensitivity. In this study, four crRNAs were designed and prepared using the oligonucleotides by in vitro transcription. The cleavage efficiency induced by Cas12a-crRNA-target complex was evaluated. Figure 4a, b showed that the efficiency of Cas12a trans cleavage mediated by crRNA1, crRNA2-2, and crRNA3 had a significant statistical difference in fluorescence intensities compared with the negative group, indicating that crRNA1 without PAM sequence can also activate the Cas12a cleavage activity with prominent specificity, and thus eliminate the requirement of PAM sequence limitation. However, previous studies indicated that PAM sequence is a key factor in nonspecific collateral cleavage of Cas12a to target dsDNA, and thus the crRNA1 without PAM sequence may increase the uncertainty of detection system [23]. Interestingly, the crRNA2-2-mediated group exhibited the fastest fluorescence response compared with other crRNA-induced groups, and the fluorescence signal saturated at 3 min. In addition, crRNA-mediated conversion of a green fluorescent signal to blue fluorescent signal in the crRNA1, crRNA2-2 or crRNA3-treated group was observed under a UV light illuminator, compared to the crRNA2-1-treated and no-crRNA treated group (Fig. 4c), and the crRNA2-2-mediated group produced intense blue fluorescence under UV irradiation compared with other crRNA-treated groups. Therefore, crRNA2-2 was used in the subsequent RT-RAA-Cas12a experiments.

### Optimization of crRNA2-2 concentrations for RT-RAA-Cas12a-mediated fluorescence and LFS assay

**Fig. 5:**
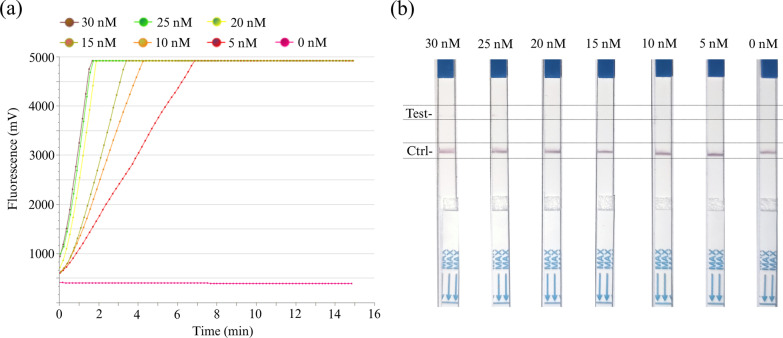
The effect of various crRNA2-2 concentrations on the performance of RT-RAA-Cas12a-based assay. **a** The RT-RAA-Cas12a-based fluorescence readouts were measured after incubation with 0, 5, 10, 15, 20, 25 and 30 nM of crRNA2-2 for 15 min and 10 min at 39 °C, respectively. There was an obvious increase in fluorescence at the concentration of crRNA2-2 between 5 and 20 nM, while the fluorescence intensities remained almost constant with the further increase of crRNA2-2 from 20 to 30 nM. **b** To test whether high concentrations of crRNA2-2 could cause false positive results, different crRNA2-2 concentrations were examined using lateral flow strip (LFS) readouts only in the absence of the amplification products in RT-RAA-Cas12a-based assay. No false-positive results were found at high concentrations of crRNA2-2 such as 20 nM and 30 nM

To achieve better performance of the RT-RAA-Cas12a-mediated assay, the effect of the concentration of crRNA on the RT-RAA-Cas12a-mediated assay by fixing the ratio of crRNA2-2 to Cas12a (1:1) was investigated. As demonstrated in Fig. 5a, there was a very good linear relationship in signal intensity at crRNA2-2 concentration between 5 and 20 nM, confirming that the reaction rate of Cas12a scaled with concentration of crRNA2-2. By contrast, the fluorescence intensities remained steady with further increases in crRNA2-2 concentration between 20 and 30 nM. A previous study has demonstrated that excess Cas12a and crRNA2-2 mixture may lead to crRNA-independent cleavage [24], indicating that excess crRNA may potentially bring about an increased risk of false-positive. Hence, 20 nM of crRNA2-2 concentrations for our RT-RAA-Cas12a-mediated assay was applied for the following de tection system. Similarly, in the RT-RAA-Cas12a-mediated LFS assay, to evaluate the potential influence of the amount of crRNA2-2 on the false-positive responses only in the absence of the target amplification products, various concentrations of crRNA2-2 from 5 to 30 nM were applied, and the reaction was visualized on LFS. As expected, the assay had a quick run time of 30–40 min, and no false-positive responses were obtained with various crRNA2-2 concentrations between 5 and 30 nM (Fig. 5b), suggesting that 20 nM of crRNA2-2 concentration may be useful in the following RT-RAA-Cas12a-mediated LFS detection to ensure the high detection limit.

### Sensitivity and specificity of RT-RAA-Cas12a-mediated fluorescence and LFS assay for NOVs GII.4 or GII.17 detection

**Fig. 6:**
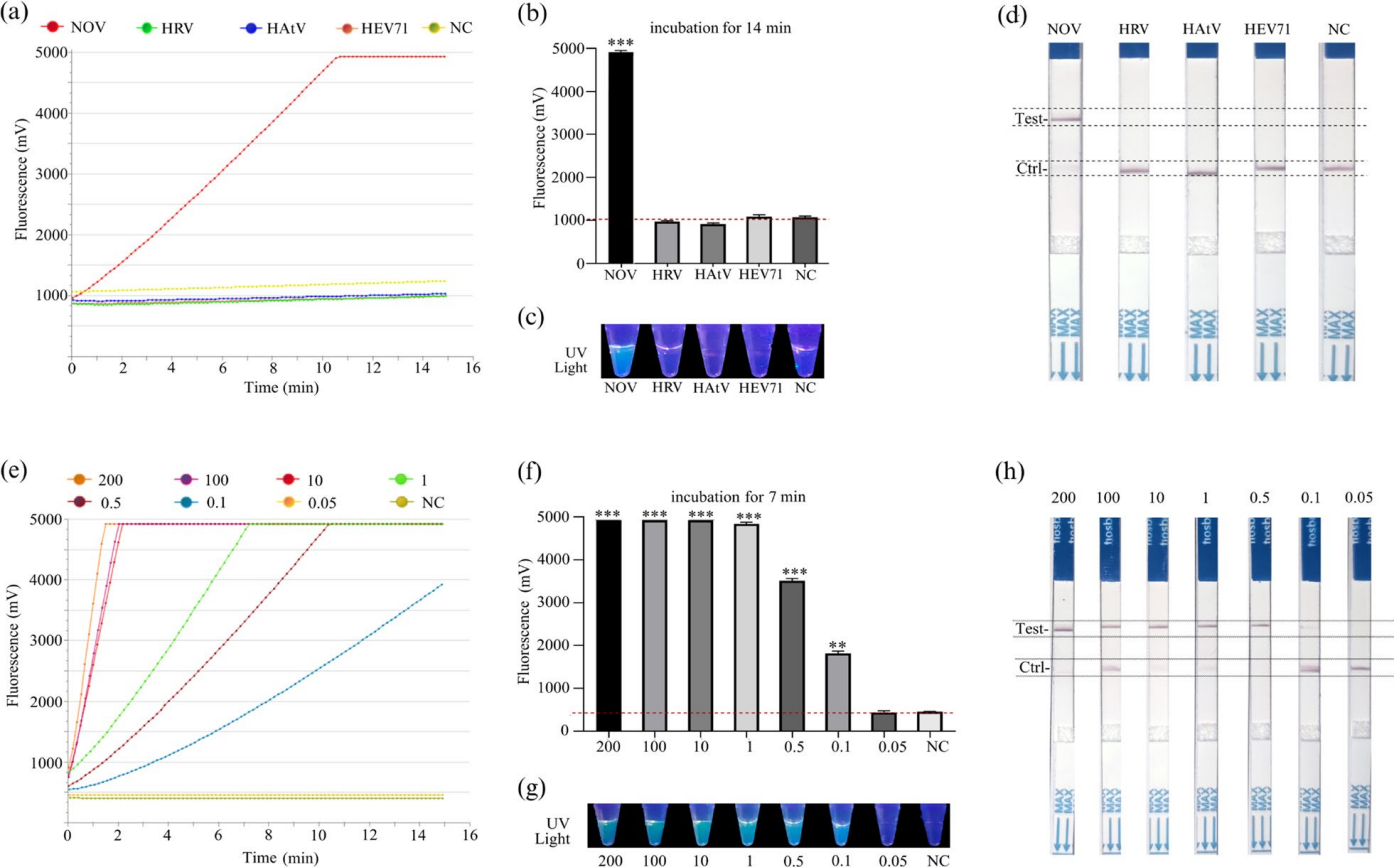
Specificity and sensitivity of the RT-RAA-Cas12a-based fluorescence and lateral flow strip (LFS) assay. **a**–**d** Specificity of RT-RAA-Cas12a-mediated real-time and end-point fluorescence and LFS assay was conducted with human rotavirus (HRV), astrovirus (HAtV), and enterovirus 71 (HEV71) at concentrations of 200 copies/μL. Visualization of Cas12a assay on NOV VP1 in real-time (**a**), end-point (**b**), under ultraviolet (UV) light (**c**) and by LFS (**d**). (**e–h**) Sensitivity was conducted with a gradient concentration of NOV standard RNA ranging from 200 to 0.05 copies/μL. Visualization of Cas12a assay on NOV VP1 in real-time (**e**), end-point (**f**), under ultraviolet (UV) light (**g**) and by LFS (**h**), respectively. Three replicates were conducted for each sample. Error bars indicate the standard deviations of three replicates (n = 3). Statistical analysis was employed to evaluate the difference between detection groups to NC. *p < 0.05; **p < 0.01; ***p < 0.001. NC, the negative control. Ctrl is the control line. Test refers to the test line

The specificity was determined using other viral genomic samples. To examine the signal generated by Cas12a when using fluorescence or lateral flow, the performance of the RT-RAA-Cas12a-mediated readout on identical products by visualizing fluorescence signals in real-time and end-point, and by lateral flow at 0, 3, 6 and 10 min was monitored. As demonstrated in Fig. 6a, the RT-RAA-Cas12a-mediated real-time fluorescence data exhibited the superior performance with high specificity without cross reactions with non-GII.4 or GII.17 targets, and was detectable within < 1 min. Similarly, the data clearly showed an efficient and specific detection of NOVs GII.4 or GII.17 when comparing end-point fluorescence after 14 min of reaction time (Fig. 6b). Specifically, the green fluorescence signal of end-point was observed under UV light while the blue fluorescence signal was observed in other samples, indicating that the visualization results were observed by the naked eye using the simple equipment (Fig. 6c), simplifying the operation of sample treatment. Meanwhile, our RT-RAA-Cas12a-mediated lateral flow strip assay exhibited high specificity for NOV GII.4 or GII.17 detection, and yielded an easy-to-interpret qualitative readout for the presence or absence of the target virus (Fig. 6d).

Next, a series of diluted NOV standard RNAs were recorded to test the analytical capability of RT-RAA-Cas12a-mediated fluorescence and LFS. `As displayed in Fig. 6e, the positive fluorescence yield was observed with a gradual enhancement as the concentration of the target RNA increased from 0.05 to 200 copies/μL. Figure 6e–g presented clearly the RT-RAA-Cas12a-mediated fluorescence could consistently detect down to ~ 0.1 copies/μL of NOVs GII.4 or GII.17 RNA targets in both real-time and end-point fluorescence detection. Moreover, the positive signal was easily visualized by the naked eye and by LFS, suggesting that RT-RAA-Cas12a mediated LFS assay exhibited a high level of sensitivity for NOVs GII.4 or GII.17 RNA. Specially, RT-RAA-Cas12a mediated LFS contributes a lot in practical application, especially for fast on-site detection. Therefore, by targeting the NOVs GII.4 or GII.17 VP1 gene, our RT-RAA-Cas12a-mediated fluorescence and LFS assay displayed a rapid, ultrasensitive and specific method for NOVs GII.4 or GII.17 detection at the nucleic acid level.

### Clinical validation of the RT-RAA-Cas12a-mediated fluorescence and LFS assay for NOVs GII.4 or GII.17 detection

**Fig. 7:**
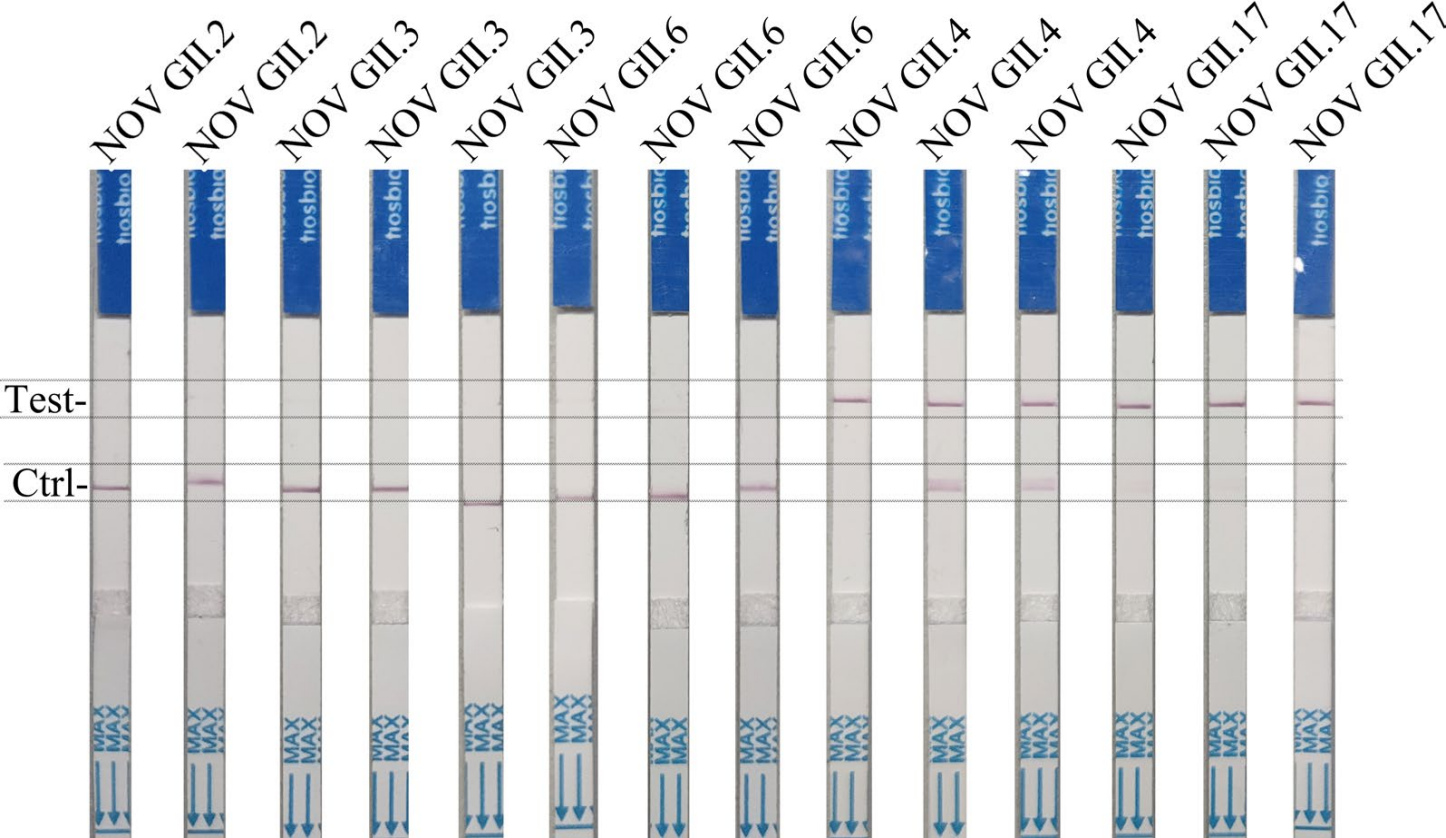
The results of representative clinical samples, including norovirus (NOV) genotype GII.2, GII.3, GII.4, GII.6 and GII.17 using RT-RAA-Cas12a-based lateral flow strip assay for NOV GII.4 or GII.17 detection

The manual RNA extraction for 80 identified clinical samples using magnetic beads processed takes about 2 h, which can practically be done using a simple magnetic separator equipment in poor-resource setting, and eliminates the need for expensive equipment. A total of 80 RNA extracts were tested in RT-RAA-Cas12a-mediated fluorescence and LFS assay. To ensure the detection accuracy, each RNA sample was measured in triple independent experiments. As demonstrated in Table 2, the results from our RT-RAA-Cas12a-mediated fluorescence and LFS readouts were highly consistent with those of RT-PCR method for detection of the NOVs in 80 total stool samples. The positive predictive agreement of Cas12a-mediated fluorescence and LFS assay for RT-RAA samples in RT-PCR assay were 95.7% and 94.3%, respectively, while the negative predictive agreement was 100%. The high concordance between our results from RT-RAA-Cas12a-mediated fluorescence and LFS readouts might be attributed to the fact that Cas12a can specifically bind to the target gene through the guidance of crRNA and activated in the presence of VP1 target genes amplified by RT-RAA, resulting in cutting of the ssDNA FQ or ssDNA FB reporter molecules mediated by Cas12a. Moreover, as shown in Fig. 7, clinical samples were assayed by RT-RAA-Cas12a-mediated LFS assay, including NOVs GII0.2, GII0.3 or GII0.6 genotypes, but only GII.4 and GII.17 were detected by LFS.

**Table 2:** Comparison of RT-RAA-Cas12a-based fluorescence and lateral flow strip (LFS) and RT-PCR of norovirus genotype GII.4 and GII.17 in 80 clinical samples

Assay	Number of samples		Detection coincidence rate with RT-PCR for 70 positive samples (%)	Detection coincidence rate with RT-PCR for 10 negative samples (%)
	Positive	Negative		
RT-RPA-Cas12a-based fluorescence	67	10	95.7	100
RT- RPA-Cas12a-based-LFS	66	10	94.3	
RT-PCR	70	10

## Discussion

In humans, norovirus GII.4 viruses have been reported to be responsible for most gastroenteric infections for more than two decades [3]. Recently, the emergence of the novel GII.17 norovirus genotype has been reported, and it replaced the pandemic strain GII.4 as the leading cause of gastroenteritis outbreaks in China and Japan since the winter season 2014 and 2015 [9]. Early diagnosis is crucial to stop the extensive spread of this virus. Currently, the reverse transcription-polymerase chain reaction (RT-PCR) represents the most routinely used method for the early detection of the pathogens; however, RT-PCR that highlights a critical need for specialized laboratory equipment and trained professionals, is not applicable to rapid POC diagnostic tests. The limitations of current detection approaches constitute serious barriers for the real-time monitoring and earlier detection of the highly infectious pathogen in low-resource settings, with the aim of reducing community and hospital transmission. Therefore, there is an urgent need for a fast, simple, inexpensive and efficient nucleic acid-based diagnostic method that is feasible for routine POC use in the low-resource setting.

In this study, we presented a simple, fast, and highly specific RT-RAA-Cas12a-mediated fluorescence and LFS assay for the detection of NOVs GII.4 and/or GII.17. Our RT-RAA-Cas12a-mediated assay combines fully an isothermal amplification of RNA samples with optimized crRNAs with high sensitivity and specificity to enable robust NOVs GII.4 and/or GII.17 detection. Notably, the detection results of the RT-RAA-Cas12a-mediated fluorescence and LFS assay can be evaluated via multiple methods, including the naked eye under UV, LFS, and real-time and end-point fluorescence. The application of real-time fluorescence-based readouts delivers highly specific, sensitive assays that are free from contamination. In addition, results interpretation based on naked eye and LFS detection is independent of sophisticated equipment and professional technicians, which provides an advantageous tool for POC and resource-poor settings. Also, in end-point fluorescence readouts, fluorescence intensities were collected during the amplification reaction, and it is possible to enable semiquantitative detection by determining end-point fluorescence data.

The RT-RAA-Cas12a-mediated LFS assay was carried out using the ssDNA FB Reporter whose both ends were labeled with FAM and Biotin [23, 25]. For the negative sample, the gold-particle-anti-FAM antibody was first sufficiently conjugated with ssDNA FB reporter, and then was captured by streptavidin located at the control band. By contrast, for the positive sample, when the ssDNA FB reporter was cleaved, the resulting free FAM was conjugated with gold-particle-anti-FAM antibody, and the complex was accumulated at the test band, and the accumulation of the complex was decreased correspondingly at the control band. Therefore, the strength of the test band depended on the cleavage efficiency, and was inversely proportional to that of the control band.

Compared to our previously reported detection method of NOV GII.4 nucleic acid based on RT-RAA-Cas12a [23], this novel assay was highly specific and ultrasensitive for the detection of NOVs GII.4 or GII.17. Interestingly, compared with our previous publication [23], in this study, the introduction of the RT-RAA-Cas12a-mediated end-point fluorescence assay may be preferable for a simple read-out by a portable, the battery-operated fluorescent reader or the handheld UV light in low-resource settings. Furthermore, the assay can generate real-time amplification result in < 15 min, which eliminates the need to open the reaction vials after the reaction, thereby simplifying the workflow and minimizing contamination risk. This ultrahigh sensitivity might be attributed to the high amplification of RT-RAA using optimal RT-RAA primers, as well as the efficient endonuclease activity of the target driven by Cas12a through the optimized concentration ratio of crRNA to Cas12a. Furthermore, given the sequence-based stringent recognition of Cas12a, this novel assay displayed a high specificity in the NOVs GII.4 and/or GII.17 detection with no cross-reaction with other viral sequences, highlighting its potential as a potent tool for disease diagnostics.

To evaluate the validity and clinical potentials of the RT-RAA-Cas12a-mediated fluorescence and LFS assay, viral RNAs extracted from NOVs in fecal samples were used, achieving highly consistent detection results with that of RT-PCR method, with 95.7% and 94.3% positive predictive agreement and 100% negative predictive agreement. The false-negative samples of RT-RAA-Cas12a-mediated fluorescence and LFS assay were selected for verification. The results showed that the false-negative results can be attributed to reagents or consumables, including the lyophilized reagent and LPS for the RT-RAA assay. Therefore, the reliability of reagents or consumables needs further improvement. Significantly, our RT-RAA-Cas12a-mediated assay can continue to reduce and streamline the operation steps with a portable microfluidic-based chamber. Specifically, by integrating air-dried specific primers, fluorescence probes, RT-RAA and Cas12a, and liquid reagents for nucleic acid extraction packaged in stick-pack, a rapid and simple sample-to-result molecular diagnostic platform can be exploited for use in low resource settings such as airports, local emergency departments and clinics [26]. Additionally, by combining the microfluidic multiplexed detection technology [27], our RT-RAA-Cas12a-mediated assay can be performed to enable multiplexed detection of sample at the same time, thereby detecting and differentiating NOVs and other pathogen infections.

## Conclusion

In sum, by combining CRISPR-Cas12a-assisted nucleic acid detection, RT-RAA-mediated isothermal amplification, and the lateral flow strip, a practical RT-RAA-Cas12a-mediated fluorescence or LFS assay was established for detection of the NOVs GII.4 and/or GII.17. Given its speed, simplicity and low-cost, the RT-RAA-Cas12a-mediated fluorescence or LFS assay holds great promise for the early detection of NoVs GII.4 and/or GII.17, especially in low-resource settings.

## Data Availability

All data generated or analyzed during this study are included in this article.
